# Phage Lysins for Fighting Bacterial Respiratory Infections: A New Generation of Antimicrobials

**DOI:** 10.3389/fimmu.2018.02252

**Published:** 2018-10-16

**Authors:** Roberto Vázquez, Ernesto García, Pedro García

**Affiliations:** ^1^Centro de Investigaciones Biológicas (CSIC), Madrid, Spain; ^2^Centro de Investigación Biomédica en Red de Enfermedades Respiratorias (CIBERES), Madrid, Spain

**Keywords:** phage lysins, pneumonia, respiratory infection, antibacterials, antibiotic resistance, endolysins

## Abstract

Lower respiratory tract infections and tuberculosis are responsible for the death of about 4.5 million people each year and are the main causes of mortality in children under 5 years of age. *Streptococcus pneumoniae* is the most common bacterial pathogen associated with severe pneumonia, although other Gram-positive and Gram-negative bacteria are involved in respiratory infections as well. The ability of these pathogens to persist and produce infection under the appropriate conditions is also associated with their capacity to form biofilms in the respiratory mucous membranes. Adding to the difficulty of treating biofilm-forming bacteria with antibiotics, many of these strains are becoming multidrug resistant, and thus the alternative therapeutics available for combating this kind of infections are rapidly depleting. Given these concerns, it is urgent to consider other unconventional strategies and, in this regard, phage lysins represent an attractive resource to circumvent some of the current issues in infection treatment. When added exogenously, lysins break specific bonds of the peptidoglycan and have potent bactericidal effects against susceptible bacteria. These enzymes possess interesting features, including that they do not trigger an adverse immune response and raise of resistance is very unlikely. Although Gram-negative bacteria had been considered refractory to these compounds, strategies to overcome this drawback have been developed recently. In this review we describe the most relevant *in vitro* and *in vivo* results obtained to date with lysins against bacterial respiratory pathogens.

## The impact of bacterial respiratory diseases on human health

Lower respiratory tract infections remain the most deadly communicable diseases, and caused 3.2 million deaths worldwide in 2015 (1). Tuberculosis is still to date among the top 10 death causes, and community-acquired pneumonia is the single largest bacterial infectious cause of death in children worldwide (2). *Streptococcus pneumoniae* (pneumococcus) accounts for most of the bacterial pneumonia cases in children, followed by *Haemophilus influenzae* type b, and other bacterial pathogens: *Streptococcus pyogenes* (group A *Streptococcus*), non-typeable *H. influenzae, Staphylococcus aureus, Mycoplasma pneumoniae, Moraxella catarrhalis*, and *Klebsiella pneumoniae* (3). Pneumococcus is also a common cause of community-acquired pneumonia in elderly patients with comorbidities (4). On the other hand, hospital-acquired pneumonia and ventilator-associated pneumonia are among the leading nosocomial infections worldwide, with an increasing frequency of multidrug resistant (MDR) Gram-negative bacteria (G–) as the bacteriologic cause (5).

Indeed, antimicrobial resistance (AMR) and associated morbidity and mortality have been increasing globally. A recent study estimated that AMR could produce 10 million deaths a year by 2050 (6), although this prediction should be taken with care (7). Accordingly, economic simulations predict that the world will suffer an annual shortfall loss of between $1 and $3.4 trillion by 2030 because of AMR (8). In this scenario, the World Health Organization (WHO) has called for global action on AMR (9). This has encouraged several actions: (a) prevention and control actions in healthcare facilities (10); (b) widespread antimicrobial stewardship programs (11); (c) reduction of antibiotic use in livestock production and the environment (12); and (d) the search for alternatives to the currently used antibiotics (13), particularly against a group of MDR bacteria having a global impact (14). Among these priority pathogens, *S. pneumoniae, H. influenzae* and those referred to as “the ESKAPE bugs” (15), are of particular concern. Of note, *Mycobacterium tuberculosis* was not included in the above list as it is already in a globally established priority for which innovative new treatments are urgently needed (16). A few decades ago, phage therapy revived as an alternative to conventional antibiotics and, since the beginning of twenty-first century, phage lytic enzymes have also been extensively tested as antibacterials. This area of research is the focus of this review and the most relevant results of certain enzymes against respiratory pathogens will be discussed. Extensive details on the issue can be found in other recent reviews (17–26).

## General characteristics of lysins

Endolysins, or more simply lysins, are phage-encoded enzymes capable of hydrolyzing the bacterial cell wall (CW) and that are synthesized at the end of the phage replication cycle. The peptidoglycan (PG) polymer is the basic component of the CW, and is composed of chains of a disaccharide repeat made up of *N*-acetylglucosamine and *N*-acetylmuramic acid, linked by β(1 → 4) glycosidic bonds. Glycan strands are cross-linked by tetra/pentapeptide side stems attached to muramic acid residues through amide bonds. Lysins are usually classified as glycosidases [glucosaminidases, transglycosylases, and lysozymes (or muramidases)], if they break any of the bonds of the glycan chain, *N-*acetylmuramoyl-L-alanine amidases (NAM-amidases), if they break the amide bonds between the glycan strands and peptide chains, or endopeptidases if they hydrolyze different bonds within peptide chains. When purified lysins are added exogenously, their CW-degrading activity can lead to rapid osmotic lysis and bacterial death. The enzymatic activity of lysins was the basis for their exploration as antibacterial agents and they were also named “enzybiotics” (27). Lysins possess several advantages over antibiotics: (a) they rapidly kill bacteria, practically upon contact; (b) they can be specific to the target pathogen, particularly against Gram-positive (G+) bacteria (28–31), which allows to preserve the normal microbiota (32); (c) development of resistance seems very unlikely (33, 34), probably because these enzymes directly target an essential and well-conserved structural component such as the PG, which cannot be easily modified without compromising fitness (35); (d) with few exceptions (36, 37), lysins are active independently of the bacterial physiological state (38, 39); (e) they are effective against MDR bacteria (20, 34, 40–42); (f) they can act synergistically with other lysins or antibiotics and thus theoretically reduce the development of resistance while increasing therapeutic efficiency; and (g) lysins are also effective killing colonizing pathogens growing on mucosal surfaces and/or in biofilms (Tables 1, 2).

**Table 1:** Selected lysins active against Gram-positive bacteria and acid-fast mycobacteria.

**Species**	**Lysin/phage**	**Susceptible bacteria tested**	**Methodology used**		**Acc. No.; comments**	**References**
			***In vitro***	***in vivo***		
***S. pneumoniae***						
	Pal/Dp-1	Pneumococci and relatives	Biofilm; synergy with Cpl-1	Colonization and sepsis (mice)	O03979	(40, 43–45)
	Cpl-1/Cp-1	Pneumococci and relatives	Biofilm; synergy with Pal and antibiotics; cell culture	Colonization, otitis, pneumonia, sepsis (mice)	P15057	(43–52)
	LytA	Pneumococci and relatives	Biofilm	Sepsis (mice)	P06653; major autolysin	(45, 53)
	Cpl-7/Cp-7	Streptococci; other G+	Biofilm		P19385	(45, 53)
	Cpl-7S	Streptococci; other G+	Cell culture	Colonization (mice), pneumococcal infection (zebrafish)	Engineered protein	(51, 53)
	Cpl-711	Pneumococci and relatives	Biofilm; synergy with antibiotics; cell culture	Colonization and sepsis (mice), pneumococcal infection (zebrafish)	Chimera of Cpl-7 and Cpl-1	(41, 51, 54)
	PL3	Pneumococci and relatives	Biofilm	Pneumococcal infection (zebrafish)	Chimera of Pal and LytA	(38)
***S. pyogenes*** **(GAS)**						
	PlyC/C_1_	GAS and other streptococci	Biofilm; cell culture (intracellular killing of GAS)	Colonization (mice)	J7M5V6	(27, 55–57)
	PlyPy/MGAS315 prophage	GAS and other streptococci		Sepsis (mice)	AAM79913	(58)
***S. agalactiae***** (GBS)**						
	PlyGBS	GAS, GBS and other streptococci		Colonization (mice)	Q5MY96	(59, 60)
***S. aureus***						
	Lysostaphin	Staphylococci	Biofilm; synergy with LysK; CHAP_K_ and antibiotics; controlled release	Sepsis and colonization (mice, rats)	P10547; from *S. simulans*	(35, 61–72)
	LysK/K	Staphylococci	Biofilm; complex with polycationic peptides		Q6Y7T6	(61, 73, 74)
	CHAP_K_	Staphylococci	Biofilm; synergy with lysostaphin; controlled release	Colonization (mice)	CHAP domain of LysK	(64, 75–78)
	ClyS	Staphylococci	Synergy with oxacillin and vancomycin	Colonization and septicemia (mice)	Chimera of Twort phage lysin (O56788) and φNM3 phage lysin (Q2FWV2)	(79)
	SAL-1/SAP-1	Staphylococci	Biofilm	Bacteremia (mouse), toxicity and pharmacokinetics (rats, dogs, monkeys), pharmacokinetics and pharmacodynamics (healthy humans)	SAL200 is a drug formulation of SAL-1	(80–84 ]
	P128	Staphylococci	Biofilm; cell culture; synergy with antibiotics	Colonization and sepsis (rats)	Chimera of Gp57 (Q6Y7R1) and lysostaphin; under clinical testing	(85–92)
	LysGH15/GH15	Staphylococci	Biofilm	Sepsis and pneumonia (mice)	D6QY02	(93–97)
	CF-301 (PlySs2)/*S. suis* 9/1591 prophage	*S. aureus, S. pyogenes, S. pneumoniae*; other G+	Biofilm; synergy with antibiotics	Sepsis (mice)	M1NS67; under clinical testing	(33, 98, 99)
	ClyF	Staphylococci	Biofilm	Sepsis (mice)	Chimera of Ply187 (O56785) and PlySs2	(100)
***Mycobacterium*** **sp**.						
	LysB/Ms6	Mycobacteria	Growth inhibition with surfactants		Q9ZX49; esterase	(101, 102)
	LysB/Bxz2	Mycobacteria	Growth inhibition with surfactants		Q9FZR9; esterase	(101)
	LysA/BTCU-1	Mycobacteria	Cell culture		O64203; intracellular killing of *M. smegmatis*	(103)
	LysB/BTCU-1	Mycobacteria	Cell culture		R9R591; intracellular killing of *M. smegmatis*; esterase	(103)

**Table 2:** Selected lysins active against Gram-negative bacteria.

**Species**	**Lysin/phage**	**Susceptible bacteria**	**Methodology used**	**Acc. No.; comments**	**References**
***P. aeruginosa***
	Lys1521/*B. amyloliquefaciens* phage	G–	Activity on intact bacteria	Q94ML9	(104–107)
	EL188/EL	G–	Activity on permeabilized bacteria	CAG27282	(108–110)
	KZ144/φKZ	G–	Activity on permeabilized bacteria	AAL83045	(108, 110)
	OBPgp279/OBP	G–	Activity on intact bacteria	YP_004958186	(111)
	Art-175	G–	Activity on intact bacteria	Chimera of KZ144 and SMAP-29 peptide	(34, 112)
	LysPA26/JD010	G–	Activity on intact bacteria, biofilm	A0A1V0EFL1	(113)
***A. baumannii***
	LysAB2/ΦAB2	G– and *S. aureus*	Activity on intact bacteria *in vivo*: sepsis (mice)	F1BCP4	(114, 115)
	LysABP-01/ØABP-01	G–	Activity on intact bacteria; synergy with colistin	KF548002	(116)
	PlyAB1/Abp1	*A. baumannii*	Activity on intact bacteria	YP_008058242	(117)
	PlyF307/RL-2015	*A. baumannii*; otros G–	Activity on intact bacteria, biofilm *in vivo*: sepsis (mice)	AJG41873	(36, 118)
	LysAB3/*A. baumannii* ATCC 17978 prophage	*A. baumannii*	Activity on intact bacteria	ABO12027	(119)
	LysAB4/*A. baumannii* ATCC 17978 prophage	*A. baumannii*	Activity on intact bacteria	CP000521	(119)
***E. coli***
	Lysep3/Ep3	*E. coli, P. aeruginosa*	Activity on permeabilized bacteria	A0A088FRS5	(120)
	Lysep3-D8	G–, *Streptococcus* sp.	Activity on intact bacteria	Chimera of Lysep3 and Lys1521 (Q94ML9)	(121)
	Colicin-lysep3	*E. coli*	Activity on intact bacteria *in vivo*: intestinal infection	Chimera of Lysep3 and colicin A (Q47108)	(122)
	EndoT5/T5	*E. coli*	Activity on permeabilized bacteria	Q6QGP7	(123)
	PlyE146/*E. coli* 8.0569 prophage	G–	Activity on intact bacteria	EKK47578	(37)
***K. pneumoniae***
	K11gp3.5/K11	G–	Activity on permeabilized bacteria	B3VCZ3	(124)
	KP32gp15/KP32	G–	Activity on permeabilized bacteria	D1L2U8	(124)
	KP27 lysin/KP27	G–	Activity on permeabilized bacteria; cell culture	K7NPX3	(125)
***C. freundii***
	CfP1 lysin/CfP1	*Citrobacter* sp.	Activity on intact bacteria	A0A1B1IXL3	(126)
***S. maltophilia***
	P28	G– and some G+	Activity on intact bacteria	Lytic enzyme from a bacteriocin system	(127)
***Burkholderia*** **sp**.
	AP3gp15/AP3	G–	Activity on permeabilized bacteria	A0A1S5NV50	(128)

Lysins encoded by phages infecting G+ bacteria generally display a modular structure, comprising one or more catalytic domains (CDs) and one or more CW binding domains (CWBD). Although the species specificity of a lysin is generally assigned to its CWBD, there are some data suggesting that combined interactions of CD and CWBD with unknown CW receptors may play a significant role (129). On the other hand, phages from G– bacteria usually encode globular lysins with a single CD, with several exceptions (31, 111, 128).

Concerning their systemic, therapeutic use, it has been alleged that lysins, as foreign proteins, could be expected to trigger the production of neutralizing antibodies that might hinder their antibacterial action in subsequent administrations. However, early studies addressing this potential drawback, strongly suggested that highly immune serum slows down—but does not block—lysins (46, 130). Pre-clinical and clinical trials with the antistaphylococcal lysin SAL-1 have been performed in animal models and, lately, in humans. An immune response was indeed elicited after repeated intravenous injections of SAL200, as demonstrated by the presence of specific antibodies and reduced C3 complement levels in the animal blood samples (80). Still, pharmacokinetic, pharmacodynamic, and tolerance studies of SAL200 in monkeys and humans did not show any serious adverse effects or clinically significant alterations even at the highest dose tested (81, 82). Anyhow, host immune responses to specific lysin formulations must always be considered concerning safety and improving the therapeutic potential of lysins.

The antibacterial efficacy of lysins can be improved by several means including: (a) replacement of certain amino acids to modify the net charge of the enzyme (53, 131) or allow dimerization (132); (b) deletion of entire domains (75, 133); (c) construction of chimeric proteins by domain shuffling (41); (d) fusion to cationic peptides (or other domains) to render lysins capable to cross the outer membrane (OM), a widely recognized drawback of lysin therapy against G– bacteria (122, 134, 135), or to increase CW affinity (136); (e) co-administration of lysins with membrane destabilizing agents (EDTA, carvacrol, etc.), especially in G– pathogens (53, 112).

## Lysins against gram-positive bacteria

### Streptococcus pneumoniae

The key aspect of the *S. pneumoniae* system is the role of the aminoalcohol choline in the enzymatic activity of the bacterial autolysin LytA, and the pneumococcal phage lysins. Choline forms part of the (lipo)teichoic acids and constitutes an absolute requirement for the binding of these enzymes—members of the choline-binding family of proteins (CBPs) (137)—to the CW substrate. This peculiarity explains the extreme specificity of CBPs for pneumococci. The first article reporting the use of a CBP as an enzybiotic demonstrated the capacity of the NAM-amidase Pal to kill pneumococci of every serotype tested, including penicillin-resistant isolates (40). These results were confirmed in a mouse model of nasopharyngeal carriage (27). The Cpl-1 lysozyme has also been successfully tested in several *in vitro* assays and in different animal models of infection (46–48), and a synergistic effect was found when Cpl-1 was used together with several antibiotics (49, 50), or in combination with Pal (43, 44). The Cpl-7 lysozyme represents an exception to choline-recognizing pneumococcal lysins, since it harbors a different CWBD (138–140) that allows it to recognize and kill a broader range of bacteria. Moreover, the bactericidal effect of Cpl-7 has been improved in the engineered Cpl-7S by inverting the net charge of its CWBD (53). To date, the most powerful killing lysins tested against *S. pneumoniae* are nonetheless chimeric proteins: Cpl-711, a chimera of Cpl-7 and Cpl-1 (41), and PL3, a fusion protein between Pal and LytA [Table 1; (38)]. Treatment with Cpl-711 strongly reduced the attachment of *S. pneumoniae* to human epithelial cells, and a single intranasal dose of Cpl-711 significantly reduced nasopharyngeal colonization in a mouse model (51).

### Staphylococcus aureus

Although *S. aureus* is frequently carried asymptomatically in humans, it is also the cause of a variety of diseases and, particularly, methicillin-resistant strains (MRSA) are responsible for a great percentage of all infections, up to 80% in some countries (141). The *S. aureus* PG displays a characteristic pentaglycine interpeptide cross-linking the glycan strands (142). Most tested lysins in the *S. aureus* system contain two CDs (endopeptidase and NAM-amidase) together with an SH3b CWBD (61, 143, 144). Although the exact interaction between the CWBD and the structures to which these domains bind remains to be demonstrated in many cases, it has been proposed that some CWBDs recognize the pentaglycine peptide cross-bridge (145) or the CW-associated glycopolymers (79). Of note, the vast majority of studies reporting the therapeutic use of lysins are directed to fight *S. aureus* infections (20, 21). Together with lysostaphin (produced by *Staphylococcus simulans*), LysK and its derivatives seem to be the most lethal lysins against *S. aureus*, including MRSA (73, 76, 146, 147) as well as vancomycin-intermediate and -resistant isolates [see reference (21) and references therein]. Other examples of anti-staphylococcal lysins include several engineered proteins such as chimeric or truncated proteins (76, 85, 100, 148, 149) or fusion proteins with short cationic peptides able to cross the eukaryotic membrane and kill intracellular *S. aureus* (150, 151). Nevertheless, lysin-based studies that consider *S. aureus* as a respiratory pathogen are scarce and only include some decolonization assays (62, 63, 75, 85) and a single example of endolysin efficacy in a mouse *S. aureus* pneumonia model (93).

### Other gram-positive pathogens and mycobacteria

*S. pyogenes* is a major causative agent of upper respiratory tract infections (152). The most relevant example of a lysin targeting this pathogen is PlyC, a peculiar multimeric enzyme that kills group A streptococci with high efficiency (27, 55). In addition, the ability of PlyC to penetrate respiratory tract epithelial cells to eliminate intracellular *S. pyogenes* cells has also been proven (56). This intracellular activity overcomes one of the major drawbacks of antibiotic therapy against streptococcal throat infections, which is bacterial self-protection by cellular invasion. Other lysins reported to kill *S. pyogenes* are PlyPy (58) and the broad range, pneumococcal phage-derived Cpl-7S (53). Besides, group B streptococci are known to cause severe pneumonia in newborns (153). At least one attempt has been conducted in mice toward oropharyngeal decolonization of group B streptococci using PlyGBS lysin (59).

The acid-fast *M. tuberculosis* is still rather unexplored for the development of lysin-based therapy. This might be due to the peculiarity of *Mycobacterium* CW structure, which comprises a thick PG layer covalently attached to arabinogalactan sterified with mycolic acids (154). Because of this architecture, the lytic cassette of mycobacteriophages comprises two different lytic enzymes: a classical PG hydrolase (usually named LysA) and mycolyl-arabinogalactan esterase (LysB), which cleaves the ester bond linking mycolic acid to the arabinogalactan-PG layer. As a result, the mycolic acid layer detaches from the cell, rendering vulnerable to osmotic shock and, finally, lysis (155). Some *in vitro* assays have been conducted with both mycobacteriophage-derived hydrolases, yielding, in general, promising results that show either growth arrest (101) or a bactericidal effect (103), but further research is still required. The mycobacterial endolysins and their therapeutical potential have been recently reviewed (156).

## Lysins against gram-negative bacteria

### Pseudomonas aeruginosa

The first lysins tested against *P. aeruginosa*, for example, EL188, only killed bacteria when membrane permeabilizers (e.g., polycationic agents, EDTA) were co-administered (108, 109). Due to the potential difficulties of therapies based on the co-administration of lysins and permeabilizing agents, some of the most recent efforts have been directed toward the engineering of the enzymes themselves, giving rise to the “artilysin” concept (134). In this study, lysins were fused to cationic, antimicrobial peptides (AMPs), and these fusions were able to exert a permeabilizing activity that allowed them to cross *P. aeruginosa* OM to degrade the PG layer both *in vitro* and *in vivo* (134). Art-175 is an artilysin that was constructed by fusing lysin KZ144 and the sheep myeloid AMP 29 (SMAP-29), and further optimizing the thermostability of the resulting chimera by point mutation of several cysteine residues (34). Art-175 was able to efficiently kill either antibiotic-susceptible or MDR *P. aeruginosa* strains. Of note, Art-175 also controlled the appearance of persisters, i.e., bacterial subpopulations transiently tolerant to antibiotics that often appear upon antiinfective chemotherapy (157).

Despite the engineering efforts mentioned above, lysins able to lyse G– bacteria on their own are also currently available. Typically, this intrinsic activity from without relies on non-enzymatic mechanisms, which were first described for the T4 phage lysozyme (158) and then in several *P. aeruginosa* phage lysins (159). These lysins harbor AMP-like elements (peptides with an amphipathic secondary structure and a positive net charge) that destabilize the OM. In some cases, as for T4 lysozyme, these regions account for the bactericidal activity of the enzyme to a higher extent than the enzymatic activity itself (158). One of the first examples of a lysin with a natural cationic peptide exploited as an enzybiotic was the *Bacillus amyloliquefaciens* phage lysin Lys1521, which was indeed able to lyse *P. aeruginosa* cells (104). Other examples of *P. aeruginosa* lysins with intrinsic anti-G– activity include OBPgp279 (124) and LysPA26 (113). Although active research is being performed to deal with the OM barrier issue, no extensive *in vivo* experimental evidence has been provided for the clearance, upon lysin treatment, of *P. aeruginosa* from respiratory infections.

### Acinetobacter baumannii

In general, lysins against G– bacteria appear to be less specific than their G+ counterparts, possibly due to the (apparently) simpler organization of the former sacculi (160). This broader spectrum allows some lysins to kill several pathogenic genera, like the already mentioned lysin LysPA26, which besides *P. aeruginosa* can also lyse other G– pathogens such as *E. coli, K. pneumoniae* or *A. baumannii* (113), or Art-175, which also kills *A. baumannii* (112). This bacterium is a potential respiratory pathogen (particularly for immunocompromised and debilitated patients) that is receiving great attention in recent years due to its worrisome increased antibiotic resistance (161). Thus, several enzybiotics have also been developed with emphasis in their *A. baumannii* killing capacity, such as LysAB3 and LysAB4 (119), PlyAB1 (117), and LysABP-01 (116).

PlyF307 was capable of killing *A. baumannii* isolates, including MDR strains, both in planktonic and biofilm cultures (36) and represents the first example of an intact lysin with intrinsic anti-G– activity tested in a mammalian (mouse bacteremia) model. Unsurprisingly, it was later determined that such intrinsic activity from without partly resided in a cationic peptide located in the C-terminal domain of the lysin (118). Further studies revealed that this region contains sub-domain structural motifs with membrane permeabilizing ability, but lacking enzymatic activity; similar motifs have also been found in other lysins. For example, lysin LysAB2 (114) represents a broad-spectrum enzybiotic, both active against G+ and G– bacteria (*A. baumannii, Escherichia coli* and, surprisingly, *S. aureus*). Based on its permeabilizing properties (114), AMPs based on the C-terminal region of LysAB2 have been synthesized and demonstrated high antimicrobial activity when tested in mice infected with *A. baumannii* (115).

### Other gram-negative pathogens

In spite of being a prominent member of the ESKAPE group (162), there are only few reports of lysins active against *K. pneumoniae*. As already mentioned, LysPA26 also showed bactericidal activity against *K. pneumoniae* (113). Consequently, it is conceivable that some of the other broad spectrum anti-G– lysins would kill *K. pneumoniae*. As for specific *Klebsiella* phage lysins, some examples of lysins with proven lytic activity are those from phages K11, KP32, and KP27 (124, 125, 163), but only KP32 and KP27 were tested for their anti-*Klebsiella* activity. Although usually associated with intestinal infections, *E. coli* is also a frequent cause of nosocomial pneumonia (164). Again, some of the other G– lysins are also active against *E. coli* (105, 113, 114, 116, 124). Specifically from an *E. coli* phage, Lysep3 lysin has demonstrated noticeable activity against permeabilized *E. coli* cells (120). Moreover, a chimeric construction between Lysep3 and a colicin was able to traverse the OM via specific recognition by OM transporters (122, 165).

## Concluding remarks and future trends

As MDR bacterial respiratory pathogens are increasingly prevalent, alternative therapeutics are urgently needed. Lysins represent more than a hope in this scenario and may be a perfect counterpart to therapies based on standard antibiotics. The potential for lysin development is seemingly endless. For example, thousands of putative lysins, many of which displaying novel domain architectures, have been recently described using bioinformatic techniques (166). All this huge amount of information, together with the crystal structures of lysins and a more detailed knowledge on the bacterial CW structure, will provide better insights to design and construct “tailor-made lysins” potentially directed against any desired pathogen. Drug delivery and other added-value systems involving lysins are now also being researched by setting up different approaches (167–170). Several polymers have been studied as potential drug release vehicles not only for research but also for clinical purposes. Particularly interesting is the case of poly(*N*-isopropylacrylamide) (PNIPAM) that has been used for the coadministration of the CHAP_K_ lysin and lysostaphin through a thermally triggered release event (the temperature increase due to infection) (64).

Although a limited number of endolysins have entered clinical trials and some of them are already available in the market [reviewed in reference (18)], phages and phage-based products are subjected to strict regulatory measures (171). Moreover, in spite of their demonstrated specificity and lack of resistance development, the use of phage endolysins in humans raises several concerns. Among them, the relatively short plasma life of lysins, their immunogenicity and possible toxicity, the proinflammatory response to bacterial debris, and the difficulties to attack intracellular bacteria have been mentioned. Although only limited data of phage lysin interactions with the human body, e.g., pharmacokinetic/pharmacodynamic studies, have been published, it is encouraging that most (if not all) of the above mentioned potential limitations lack current experimental support (18, 23, 25). Although this scenario seems favorable toward hitting the clinic in the short term, further evidence is still due, especially when bacterial respiratory diseases—in particular, those caused by G– bacteria—are considered. Additional efforts to cover the currently unmet therapeutic requirements are warranted.

## Author contributions

RV, EG, and PG wrote, edited, and approved the final manuscript.

### Conflict of interest statement

The authors declare that the research was conducted in the absence of any commercial or financial relationships that could be construed as a potential conflict of interest.
